# Bioactivated Oxidized Polyvinyl Alcohol towards Next-Generation Nerve Conduits Development

**DOI:** 10.3390/polym13193372

**Published:** 2021-09-30

**Authors:** Elena Stocco, Silvia Barbon, Alessia Lamanna, Enrico De Rose, Annj Zamuner, Deborah Sandrin, Martina Marsotto, Alessandro Auditore, Grazia M. L. Messina, Antonino Licciardello, Giovanna Iucci, Veronica Macchi, Raffaele De Caro, Monica Dettin, Andrea Porzionato

**Affiliations:** 1 Department of Neuroscience, Section of Human Anatomy, University of Padova, 35121 Padova, Italy; elena.stocco@unipd.it (E.S.); enrico.derose@studenti.unipd.it (E.D.R.); veronica.macchi@unipd.it (V.M.); raffaele.decaro@unipd.it (R.D.C.); andrea.porzionato@unipd.it (A.P.); 2 L.i.f.e.L.a.b. Program, Consorzio per la Ricerca Sanitaria (CORIS), Veneto Region, 35128 Padova, Italy; annj.zamuner@unipd.it (A.Z.); deborah.sandrin@unipd.it (D.S.); monica.dettin@unipd.it (M.D.); 3 Department of Industrial Engineering, University of Padova, 35122 Padova, Italy; alessia.lamanna01@gmail.com; 4 Department of Physics and Astronomy “G. Galilei”, University of Padova, 35122 Padova, Italy; 5 Department of Sciences, Roma Tre University, 00154 Rome, Italy; martina.marsotto@uniroma3.it (M.M.); giovanna.iucci@uniroma3.it (G.I.); 6 Department of Chemical Sciences, University of Catania, 95124 Catania, Italy; alessandro.auditore@unict.it (A.A.); grmessi@unict.it (G.M.L.M.); alicciardello@unict.it (A.L.)

**Keywords:** oxidized polyvinyl alcohol, bioactivated scaffold, scaffold patterning, functionalization, self-assembling peptides, instructive stimuli, peripheral nerve injury, tissue engineering

## Abstract

The limitations and difficulties that nerve autografts create in normal nerve function recovery after injury is driving research towards using smart materials for next generation nerve conduits (NCs) setup. Here, the new polymer partially oxidized polyvinyl alcohol (OxPVA) was assayed to verify its future potential as a bioactivated platform for advanced/effective NCs. OxPVA-patterned scaffolds (obtained by a 3D-printed mold) with/without biochemical cues (peptide IKVAV covalently bound (OxPVA-IKVAV) or self-assembling peptide EAK (sequence: AEAEAKAKAEAEAKAK), mechanically incorporated (OxPVA+EAK) versus non-bioactivated scaffold (peptide-free OxPVA (PF-OxPVA) supports, OxPVA without IKVAV and OxPVA without EAK control scaffolds) were compared for their biological effect on neuronal SH-SY5Y cells. After cell seeding, adhesion/proliferation, mediated by (a) precise control over scaffolds surface ultrastructure; (b) functionalization efficacy guaranteed by bioactive cues (IKVAV/EAK), was investigated by MTT assay at 3, 7, 14 and 21 days. As shown by the results, the patterned groove alone stimulates colonization by cells; however, differences were observed when comparing the scaffold types over time. In the long period (21 days), patterned OxPVA+EAK scaffolds distinguished in bioactivity, assuring a significantly higher total cell amount than the other groups. Experimental evidence suggests patterned OxPVA-EAK potential for NCs device fabrication.

## 1. Introduction

A high incidence of nerve injuries and the limitations of autologous nerve supplies for grafting and nerve transfer has created interest towards the development of vanguard-engineered nerve conduits (NCs) based on smart polymers to promote nerve repair processes. Different from the first-generation devices, which were based on synthetic, non-resorbable hollow tubes (i.e., silicone- or polytetra-fluoroethylene-based) and requiring a second surgery for removal [1], the next-generation NCs, in addition to biodegradability (like the ones currently in use), should assure a controlled regeneration in axons path. To achieve this purpose, the incorporation of bioactive elements including growth factors, topographical cues and supportive cells, is the most promising strategy [2]. Experimental evidence strongly demonstrates superior outcomes in nerve repair when in the presence of such modifications, compared to clinically available devices [3] which are still far from guaranteeing acceptable regeneration results, especially in the case of large gaps (>30 mm). Thus, new materials and designs should be investigated in depth for future improvements and satisfactory clinical outcomes [4].

Recently, we described for the first time the preparation and characterization of the new polymer oxidized polyvinyl alcohol (OxPVA), distinguishing it as an intriguing material for the fabrication of biocompatible synthetic scaffolds [5], including devices to support nerve regeneration [6,7,8]. Compared to native polyvinyl alcohol (PVA), the oxidated form displays a certain biodegradation profile and interesting protein loading ability, both related to carbonyl groups present in the molecule backbone [5]. Despite encouraging pre-clinical study results showing OxPVA ability to sustain axonal regeneration, the great intrinsic potential of the polymer deserves to be explored, bearing in mind the need for optimization in conduit microenvironments [3].

To pursue this goal, OxPVA was adopted to fabricate and compare the behavior of different bioactive platforms with the perspective of achieving the principles for developing advanced and effective devices for nerve regeneration. To this purpose, two strategies were endorsed in concert: precise control over ultrastructural scaffold features (designed groove pattern) and its biochemical activation through peptide cues. Specifically, 3D printing was used to fabricate a mold with a precise pattern to impress the hydrogel surface, while the peptide IKVAV or the ionic-complementary self-assembling peptide (SAP) EAK were chosen as boosting agents to elicit neuronal cell adhesion and proliferation upon support [9]. Different functionalization approaches were also selected according to the chemical nature of the peptides: covalent binding for IKVAV, and mechanical incorporation for the SAP EAK.

The introduction in the OxPVA platform of a patterned groove is expected to encourage scaffold colonization (otherwise not possible for smooth OxPVA in vitro [5]); however, it should also increase the polymer surface available for peptide distribution and/or exposure, further optimizing OxPVA/cell interaction.

## 2. Materials and Methods

### 2.1. Peptides Synthesis

#### 2.1.1. Aoa-xx-IKVAV

The peptide Aoa-xx-IKVAV (sequence: NH_2_-O-CH_2_-CO-x-x-Ile-Lys-Val-Ala-Val-NH_2_, where x represents 7-aminoheptanoic acid residue) was obtained by solid phase peptide synthesis, using a Syro I automatic synthesizer (Multisyntech GmBH, Witten, Germany) and Fmoc chemistry. The solid support used during the synthesis was the Rink Amide mBHA resin (substitution 0.52 mmol/g; Merck KGaA, Darmstadt, Germany); we used 240 mg of resin, which was equal to 0.125 mmol (synthesis scale). Aoa was introduced as BisBoc-Aoa-OH (Merck KGaA) and the Lys side chain was protected with the Boc group (Merck KGaA). For each coupling, 5 equivalents of Fmoc-protected amino-acid, 5 equivalents of activating agent HBTU (Merck KGaA) and 10 equivalents of DIPEA (Biosolve Chimie, Dieuze, France) or Collidine (in the case of BisBoc-Aoa-OH condensation; Merck KGaA), were used with respect to the reactive groups on resin. Each single coupling lasted 45 min. Peptide cleavage from the resin, and contemporary side chain groups’ deprotection was carried out, treating the resin with 0.125 mL of MilliQ water, 0.125 mL of TES (Sigma Aldrich, Sant Louis, MO, USA) and 4.725 mL of TFA (Biosolve Chimie) at room temperature under magnetic stirring for 90 min. The crude peptide was purified in RP-HPLC (Mod. 1525 Waters, Milford, CT, USA) without the use of acetonitrile (Biosolve Chimie). The homogeneity grade (>97%) and the identity of the purified peptide were ascertained by analytical HPLC and ESI-ToF mass analysis (Mod. Mariner System 5220, Applied Biosystems, Perkin-Elmer, Waltham, MA, USA), reported in the Supplementary Materials Section (Figures S1 and S2).

#### 2.1.2. EAK

The self-assembling peptide EAK (sequence: H-Ala-Glu-Ala-Glu-Ala-Lys-Ala-Lys-Ala-Glu-Ala-Glu-Ala-Lys-Ala-Lys-NH_2_) was produced by solid phase peptide synthesis using a Syro I automatic synthesizer and Fmoc chemistry. Rink Amide mBHA resin (substitution 0.52 mmol/g) was used as the solid support. The side chain protections were OtBu for Glu and Boc for Lys (Merck KGaA). Each coupling was carried out with 5 equivalents of Fmoc-protected amino acid, 5 equivalents of HBTU and 10 equivalents of DIPEA. Each single coupling lasted 45 min. All amino acids were introduced with double couplings. Finally, the peptide was cleaved from the resin and all side chain protections were removed, through a treatment with the following solution: 0.125 mL MilliQ water, 0.125 mL TES and 4.725 mL TFA for 90 min at room temperature. The crude peptide was purified in RP-HPLC. The homogeneity grade (about 100%) and the identity of the purified peptide were ascertained by analytical HPLC and MALDI mass analysis (Mod. AB SCIEX MALDI-TOF 4800 Plus, AB SCIEX LLC, Framingham, MA, USA), reported in the Supplementary Materials Section (Figures S3 and S4).

The reaction with 5(6)-carboxytetramethylrhodamine (Merck KGaA) of the side chain-protected EAK peptide on resin produced the EAK analogue used for the SAP distribution evaluation assay. For the coupling reaction, 4 equivalents of 5(6)-carboxytetramethylrhodamine, 4 equivalents of HBTU and 8 equivalents of DIPEA were used.

### 2.2. Fabrication of the Patterned Mold-Plate

The patterned mold-plate, 120 mm × 90 mm × 2 mm (length × width × height), was designed using computer-sided design (CAD) software (Fusion 360, Autodesk, San Rafael, CA, USA). After modeling, the object was directly exported in stereolithography format (.stl) into the 3D printer slicer software (Cura software v.4.9.1, Ultimaker, Utrecht, The Netherlands), and converted into a 3D printer format (.gcode). Cura software allowed us to set the infill parameters related to the infill geometry, percentage and orientation. Specifically, a gyroid orientation with an 85% infill and 90° orientation were selected. Plate fabrication occurred through a commercial fusion filament fabrication (FFF) 3D printer (Ultimaker 2+ Connect, Ultimaker). As for the printing material, PETG (polyethylene terephthalate glycol-modified) from Ultimaker was adopted.

### 2.3. Preparation of OxPVA Solution

All reagents for polymer solution preparation were purchased by Sigma-Aldrich, unless otherwise indicated. The solution of OxPVA was prepared as previously described [5,10]. Briefly, 10 g of PVA powder [molecular weight (Mw) 146,000–186,000 Da, 99+% hydrolyzed] were suspended in MilliQ water and solubilized by heating in boiling bath under stirring for 1 h. Hence, after cooling at 37 °C, the solution underwent partial oxidation, adding 151 mg of potassium permanganate (KMnO_4_) in 10 mL of MilliQ water + 1.60 g of 70% HClO_4_ (*w*/*w*). The oxidative reaction ran for 1 h at 37 °C, up to complete discoloration of the polymer solution; thereafter, extensive dialysis occurred, using a membrane with an 8,000 Da cut-off (Sigma-Aldrich, Milan, Italy). For storage, the OxPVA polymer solution was frozen at −20 °C overnight, and then lyophilized (Speed Vac Concentrator Savant, Instruments Inc., Farmingdale, NJ, USA). For polymer recovery, 16 wt% OxPVA was weighted, suspended in MilliQ water, and then heated for 48 h at 100 °C.

### 2.4. Bioactive Scaffolds Fabrication

Different patterned scaffolds were finally compared. Specifically, functionalization with the bioactive peptides occurred through two distinct strategies: covalent binding (OxPVA-IKVAV) and mechanical incorporation (OxPVA+EAK). While covalent binding was performed after scaffold fabrication (starting from peptide-free (PF)-OxPVA scaffolds), mechanical incorporation occurred soon after lyophilized OxPVA reconstitution.

#### 2.4.1. Patterned Scaffolds Development and IKVAV Covalent Binding

To obtain the PF-OxPVA scaffolds, lyophilized OxPVA was reconstituted as previously described (see Section 2.3); once the polymer solution was cooled down to 60 °C, 3 g was poured onto a pre-heated (45 °C) glass slide (12 cm × 9 cm) and two spacers (2 mm thick) were placed at the edges of the support, with the patterned mold positioned above (motif surface in contact with the hydrogel). Thereafter, the system underwent a physical cross-linking consisting of freezing (−20 °C) and thawing (−2.5 °C) cycles (FT) of 24 h/each. After three FT cycles, the hydrogel was taken out of the mold and the discoidal scaffolds (diameter: 7 mm, thickness: 2 mm) were cut out using a sterile biopsy punch.

**Scheme 1:**
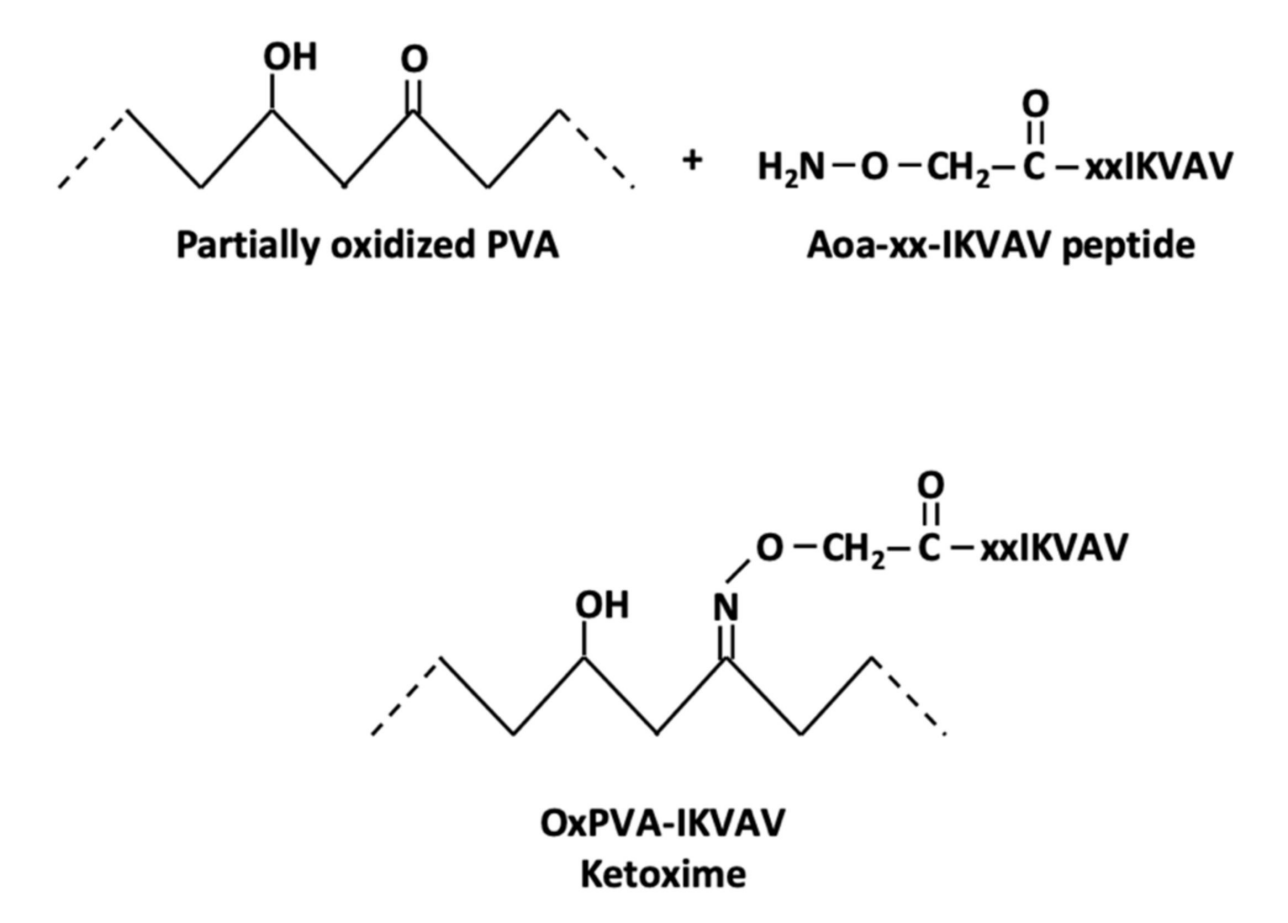
The reaction between Aoa-xx-IKVAV oxyamine N-terminal and OxPVA carbonylic groups produced OxPVA-IKVAV with ketoxime formation.

The covalent binding reaction involves the peptide oxyamine N-terminal group and the carbonylic groups of oxidized PVA, as reported in Scheme 1. OxPVA discs were inserted into a 48-multiwell plate (Corning, NY, USA), and each disk was covered with 300 µL of 1 mM Aoa-xx-IKVAV solution in an aniline acetate buffer (Sigma-Aldrich) at pH = 4.5 for 24 h at room temperature. At the end of the reaction, scaffolds were washed (one wash with buffer in an ultrasonic bath for 20 min, and three washes with MilliQ water in an ultrasonic bath for 10 min).

Patterned samples soaked in an aniline acetate buffer as described above, without the Aoa-xx-IKVAV solution, were also prepared as a control group (OxPVA without (w/o) IKVAV).

#### 2.4.2. EAK Mechanical Incorporation

Scaffolds functionalization by the SAP EAK occurred through mechanical incorporation.

The EAK powder was resuspended in MilliQ water to a final concentration of 1 mg/mL; in parallel, 3 g of OxPVA hydrogel was poured onto a glass slide as described above (see Section 2.4.1). Hence, the EAK solution was added drop by drop, to reach 0.2% *w*/*w* in OxPVA; a stainless laboratory spatula was used for the homogeneous distribution of the peptides’ solutions within the OxPVA hydrogel. Once the 2 mm-thick spacers and the patterned lamina were positioned, the system underwent FT, as previously reported.

Discoidal scaffolds (diameter: 7 mm, thickness: 2 mm) were obtained using the biopsy punch.

In parallel, patterned samples were prepared as described above, but only 0.2% *w*/*w* of MilliQ water was added drop by drop; these samples were also fabricated as a control group (OxPVA w/o EAK).

### 2.5. Scaffolds Characterization

Scaffolds were characterized for both their ultrastructure by scanning electron microscopy (SEM), and peptide functionalization method efficacy by XPS, confocal microcopy and time-of-flight secondary ion mass spectrometry, respectively.

#### 2.5.1. Ultrastructural Characterization of the Patterned Scaffolds

The ultrastructure of the patterned scaffolds was assessed by SEM. Samples were fixed with 2.5% glutaraldehyde (Sigma-Aldrich) in a 0.1 M cacodylate buffer (Sigma-Aldrich) (pH = 7.2) for 24 h, and then dehydrated through a graded ethanol series (Vetrotecnica, Padova, Italy). After critical point drying and gold sputtering, observation occurred using a Stereoscan-205 S (Cambridge Instruments, Pine Brook, NJ, USA).

#### 2.5.2. XPS Analysis of IKVAV-Functionalized OxPVA

XPS analysis was performed with a homemade instrument, consisting of preparation and analysis UHV chambers separated by a gate valve. The analysis chamber was equipped with a six-degree-of-freedom manipulator and a 150 mm mean radius hemispherical electron analyzer with a five-lens output system, combined with a 16-channel detector, giving a total instrument resolution of 1.0 eV as measured at the Ag 3d_5/2_ core level. Samples were introduced to the preparation chamber and left outgassing overnight at a base pressure of about 10^−8^ Torr, before introduction to the analysis chamber. Typical vacuum pressure in the analysis chamber during measurements was in the 10^−9^–10^−10^ Torr range. The used X-ray radiation was a non-monochromatized Mg Kα (1253.6 eV). The spectra were energy-referenced to the C1s signal of aliphatic C atoms having a binding energy BE = 285.0 eV. Atomic ratio values were calculated from peak intensities. Curve-fitting analysis of the C1s, N1s, O1s, K2p and Mn2p spectra was performed using Gaussian profiles as fitting functions, after subtraction of a Shirley-type background. When several different species were identified in a spectrum, the same fwhm value was set for all individual photoemission bands.

#### 2.5.3. Evaluation of Mechanical Incorporation Efficacy by Confocal Microscopy

To qualitatively assess the homogeneous distribution of the SAP EAK within the OxPVA scaffolds, confocal microscopy was adopted. The PF supports were used as control.

Briefly, EAK labelled with the fluorescent dye 5(6)-carboxy-tetramethyl-rhodamine (EAK-Rh) was solubilized in MilliQ water (1 mg/mL) and dispersed drop-by-drop within the OxPVA hydrogel, as reported in Section 2.4.2. After FT, the obtained scaffolds were directly analyzed through a Zeiss 800 Confocal Microscope (Zeiss, Oberkochen, DE). The excitation laser wavelength was 561 nm, while the emission signal was collected in the red channel between 580 and 620 nm. Peptide-free OxPVA scaffolds were used as the control. Three samples/group were used for the analysis.

#### 2.5.4. Time-of-Flight Secondary Ion Mass Spectrometry (ToF-SIMS) Analysis of OxPVA Functionalized with EAK through Mechanical Incorporation

Positive ion ToF-SIMS mass spectra have been acquired with a ToF-SIMS IV spectrometer (ION-TOF GmbH, Münster, Germany), using a Bi_3_^+^ analysis beam (25 keV; 0.5 pA; raster size 300 × 300 μm^2^). The primary ion fluence has been kept under 10^12^ ions/cm^2^, in order to assure static conditions.

### 2.6. Scaffolds Seeding and Bioactivity/Cytotoxicity Assessment

After scaffold fabrication, their bioactivity was evaluated through an in vitro study considering the support interaction with nervous cells from the human neuroblastoma cell line SH-SY5Y.

Preliminarily, the patterned scaffolds were adequately disinfected by soaking in 2% antibiotic/antimycotic solution (penicillin/streptomycin, Life Technologies, Paisley, United Kingdom) for 1 h under UV light (30 min/side) and then wasing 3 times in a DMEM/F-12 (1:1) basal medium (Life Technologies). Hence, they were placed in 48-well plate, seeded with SH-SY5Y cells (8 × 10^4^ cells/cm^2^; European Collection of Cell Cultures, Porton Down, UK) and cultured in a proliferative medium consisting of DMEM/F-12, supplemented with 15% FBS (Fetal Bovine Serum, Sigma-Aldrich), 1% non-essential amino acids (Sigma-Aldrich) and 1% antibiotic solution. The medium was changed every 2 days.

After 3, 7, 14 and 21 days from seeding, scaffold cytotoxicity and cell proliferation were both assessed, treating the cells with 3-(4,5-dimethylthiazol-2-yl)-2,5-dimethyltetrazoliumbromide (MTT, 0.5 mg/mL) for 4 h at 37 °C and formazan precipitates were later dissolved in 2-propanol acid (0.04 M HCl in 2-propanol). A microplate autoreader EL 13 (BIO-TEK Instruments, Winooski, VT, USA) was used to measure the optical density at 570 nm. The results were expressed as the mean number of cells grown on the seeded surface ± SD.

At 3, 7, 14 and 21 days after seeding, the cells’ adherent behavior on the scaffolds was evaluated also by SEM. Hence, the seeded supports were fixed and processed for analysis as described above (see Section 2.5.1). A Stereoscan-205 S SEM (Cambridge Instruments, London, UK) was used.

### 2.7. Statistical Analysis

Experimental data were expressed as mean ± standard deviation (SD) of six different replicates. Statistical analysis was performed by one-way analysis of variance (ANOVA) and a Tukey multiple comparison test. Differences among experimental groups were considered statistically significant with *p* < 0.05. Statistical calculations were developed through Prism 8.1.0 (GraphPad Software, San Diego, CA, USA).

## 3. Results

### 3.1. Structural and Ultrastructural Appearance of the Scaffolds

**Figure 1:**
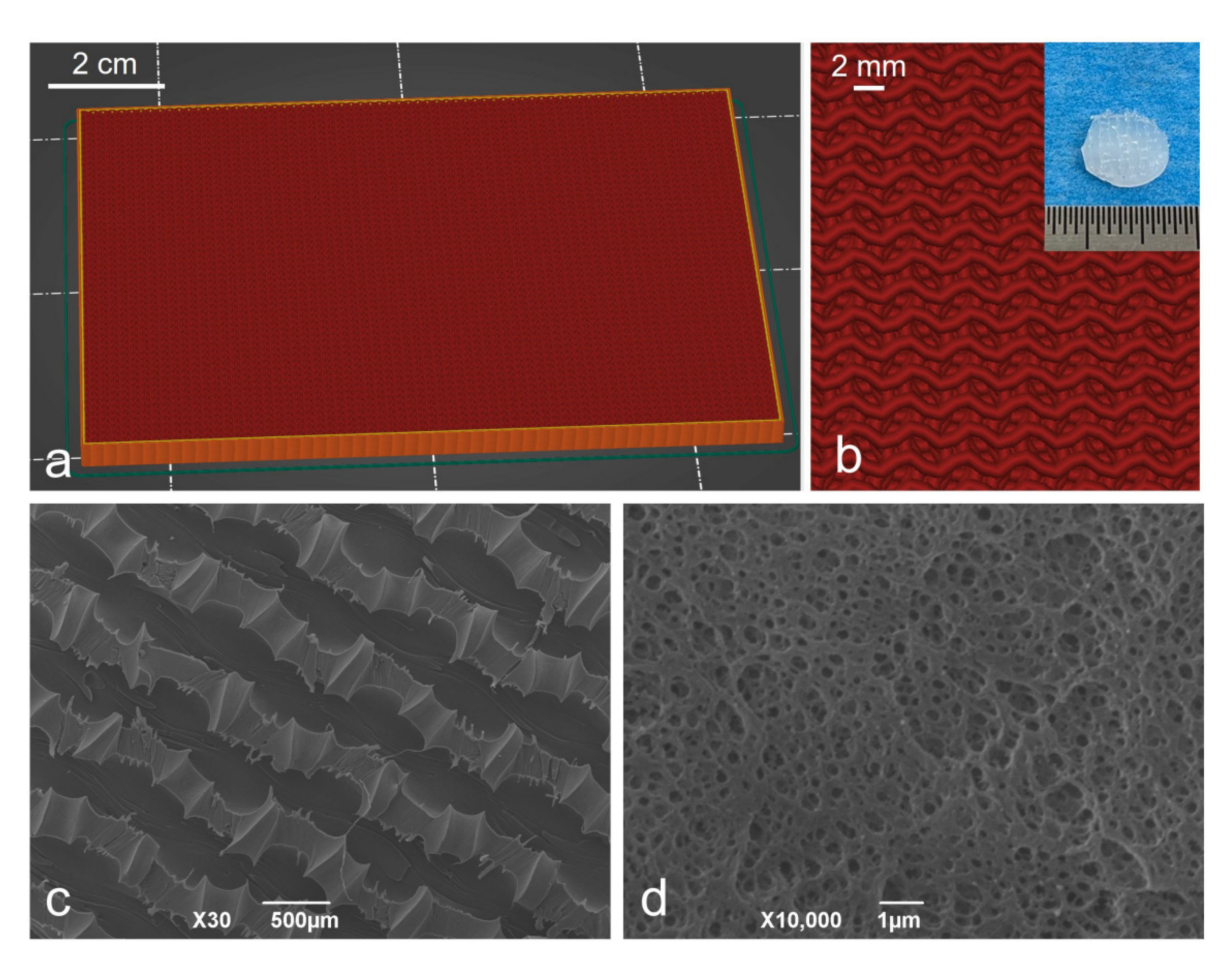
(**a**,**b**) Specific features of the 3D-printed mold designed by computer-aided design (CAD) software; (insert in **b**) representative image of scaffolds gross appearance; (**c**,**d**) representative scaffolds ultrastructure by scanning electron microscopy. Scale bars: (**a**) 2 cm; (**b**) 2 mm; (**c**) 500 µm; (**d**) 1 µm.

After freezing–thawing, the cross-linked polymers were easily removed from the molds. The membranes showed a superficial roughness which was macroscopically evident and ascribable to the presence of the pattern (Figure 1a–d).

The ultrastructure of the supports was also investigated by SEM; canalicular rows ran in parallel along the scaffolds upper surface (seeding side; Figure 1c). At higher magnification, the presence of a diffuse nanoporosity constituing a fine plot was evident (Figure 1d).

### 3.2. Peptides Distribution Analyses

#### 3.2.1. XPS Analysis of IKVAV-Functionalized OxPVA

**Figure 2:**
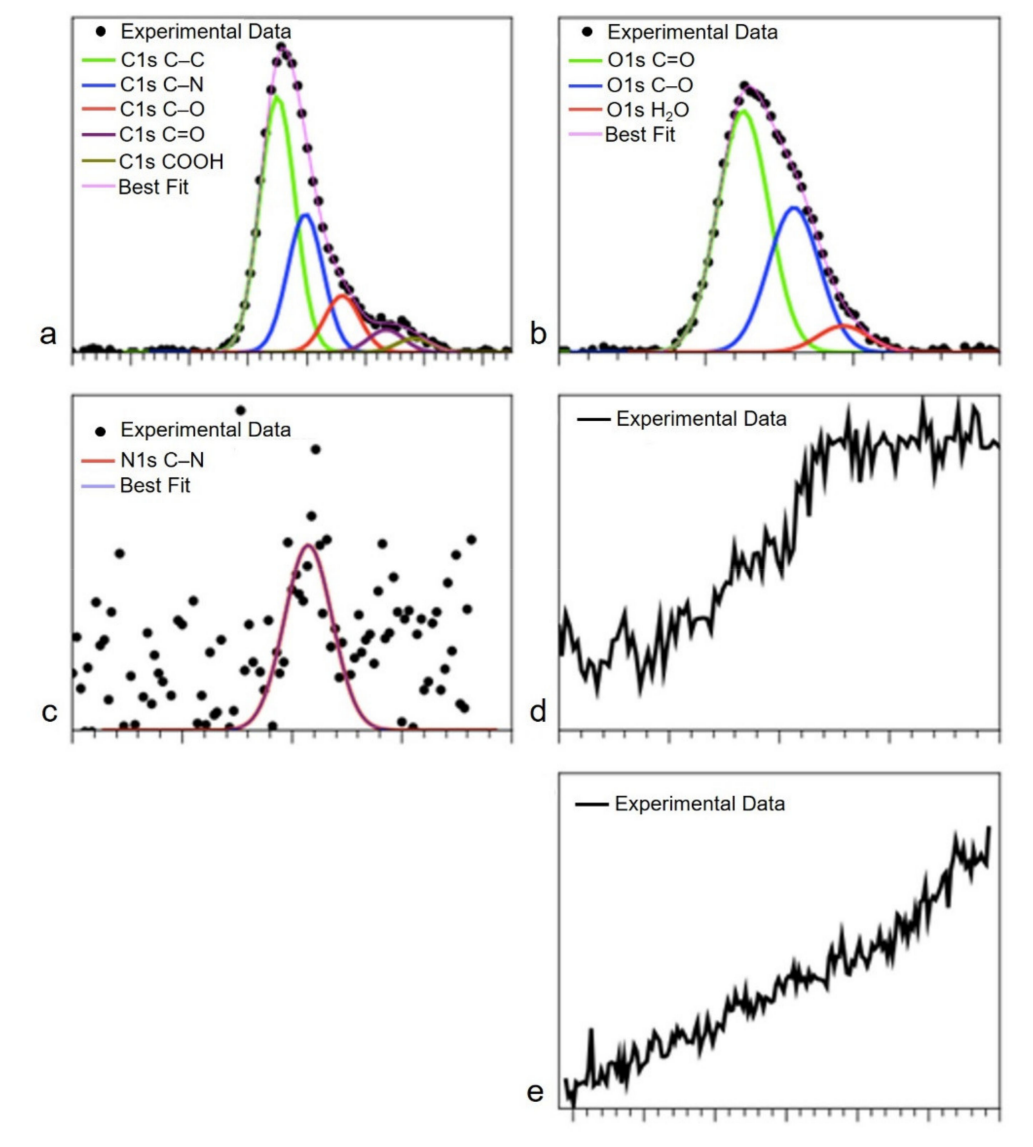
XPS spectra peak fit results of OxPVA-IKVAV. (**a**,**b**) Deconvolution of C1s (**a**) and O1s (**b**) spectra; (**c**) single component spectrum of N1s; (**d**,**e**) no significant peak in K2p (**d**) and Mn2p (**e**) spectra.

For PF-OxPVA and OxPVA-IKVAV, SR-induced XPS measurements were carried out at the C1s, N1s, O1s, K2p and Mn2p core levels. XPS data (BE, fwhm, atomic ratios) are reported in Table S1 (Supplementary Materials Section). OxPVA-IKVAV spectra are shown in Figure 2.

The C1s spectrum (Figure 2a) is made of four components. The first component, fixed at 285.0 eV, is assigned to aliphatic carbons, the second, at 286.0 eV, to C–N carbons of the immobilized peptide, the third, at 287.2 eV, is due to C–O carbons, the fourth, at 288.7 eV, to C=O carbons and the last, at 289.7 eV, is assigned to COOH carbons [Ref XPS1, XPS2]. The O1s spectrum (Figure 2b) is composed of three components. The first component, fixed at 531.3 eV, is associated with peptide oxygens, the second, at 533.0 eV, with OxPVA oxygens and the third, at 534.7 eV, with physisorbed water oxygens [11,12].

The presence of a N1s signal is clear proof of successful peptide immobilization. The N1s spectrum (Figure 2c) consists of a single component at 400.7 eV, associated with C–N peptide nitrogens [13].

K2p and Mn2p spectra do not show any significant peak (Figure 2d,e), indicating that there was no KMnO_4_ left on the sample surface.

#### 3.2.2. Homogeneous EAK-Rh Distribution within OxPVA by Mechanical Incorporation

Once incorporated within the hydrogel, drop by drop, the EAK-Rh conferred a typical pink colour to the polymer, maintained even after FT. Efficacy of the mechanical incorporation was preliminarily desumed once the membrane was separated from the mold; in fact, the colour appeared homogeneously distributed, without evident Rh clots within the cross-linked membrane.

**Figure 3:**
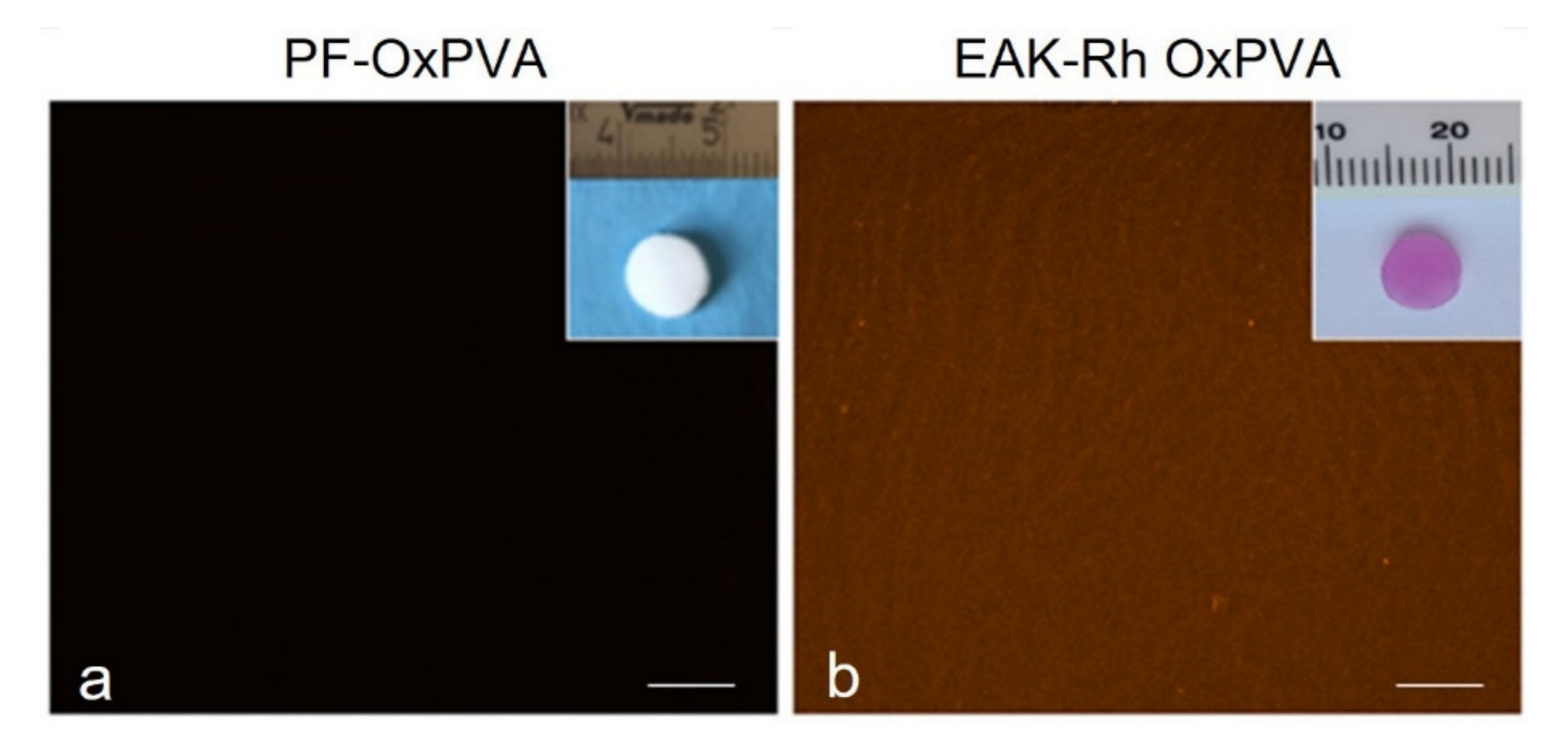
Assessment of efficacy in SAPs mechanical incorporation. The rhodamine-labeled peptide (EAK-Rh) emits a fluorescent signal (orange) collected between 580 and 620 nm when laser-excitated at 561 nm wavelength. No signal was detected in the peptide-free scaffold (PF-OxPVA) (**a**) compared to the EAK-Rh OxPVA (**b**). Scale bar: 30 µm.

Subsequently, these gross-appearance features were confirmed by confocal microscopy. The analysis showed a uniform fluorescent orange signal once the EAK-Rh was excited. In contrast, PF-OxPVA membranes did not emit a signal in the same experimental conditions, as expected (Figure 3).

#### 3.2.3. Time-of-Flight Secondary Ion Mass Spectrometry (ToF-SIMS) Analysis of OxPVA Functionalized with EAK through Mechanical Incorporation

Signal’s characteristics of PF-OxPVA and OxPVA+EAK have been identified in the ToF-SIMS spectra.

**Figure 4:**
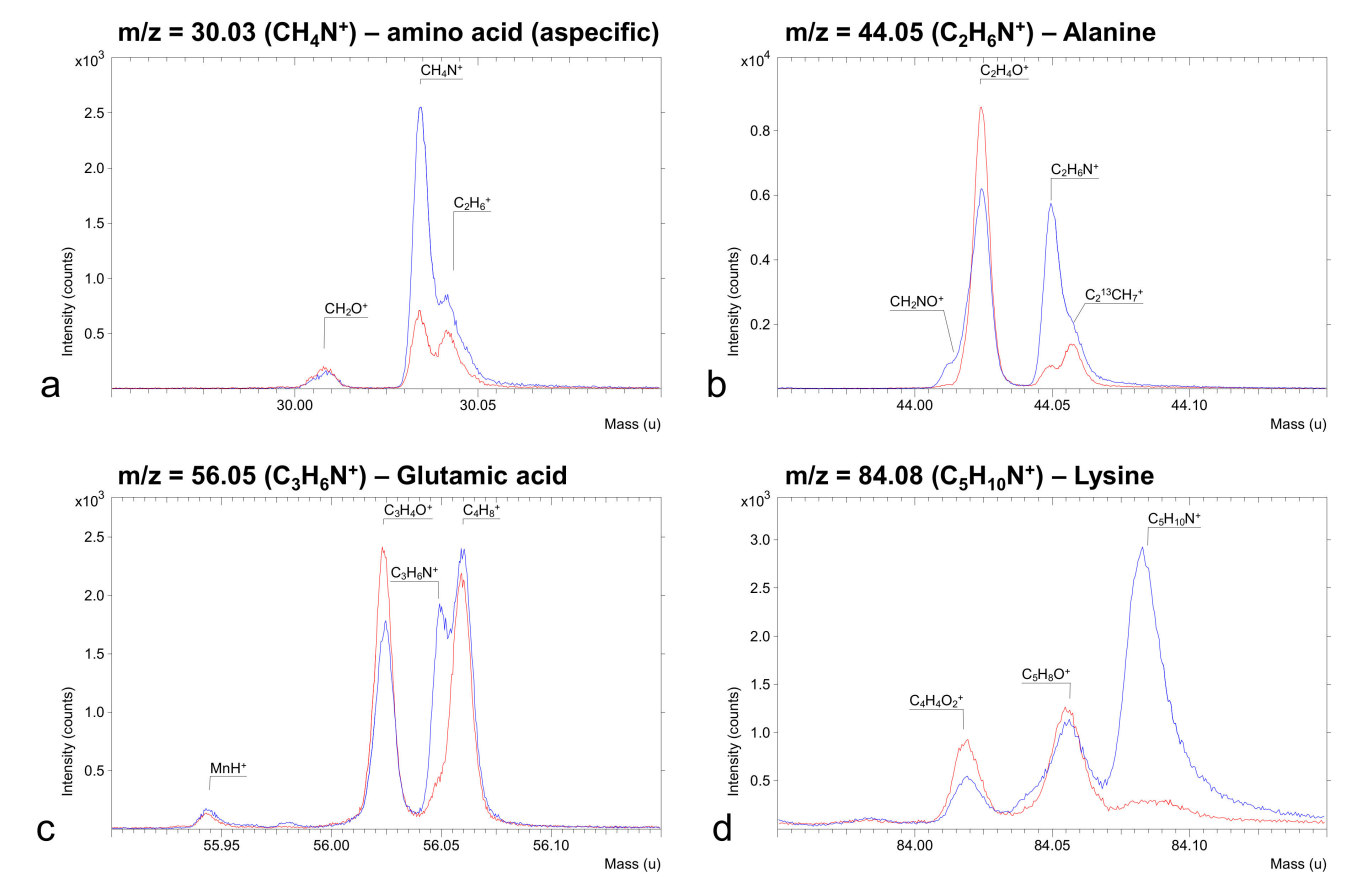
ToF-SIMS spectra of the mass region of the main fragments related to aspecific amino acid (**a**), alanine (**b**), glutamic acid (**c**) and lysine (**d**). The red line is refers to the PF-OxPVA spectrum whereas the blue line refers to the OxPVA+EAK spectrum.

The main fragmentation pattern of aspecific amino acid, alanine, glutamic acid and lysine have been recognized and identified in the sample of OxPVA+EAK. The mass region of the main fragments is reported in Figure 4.

**Figure 5:**
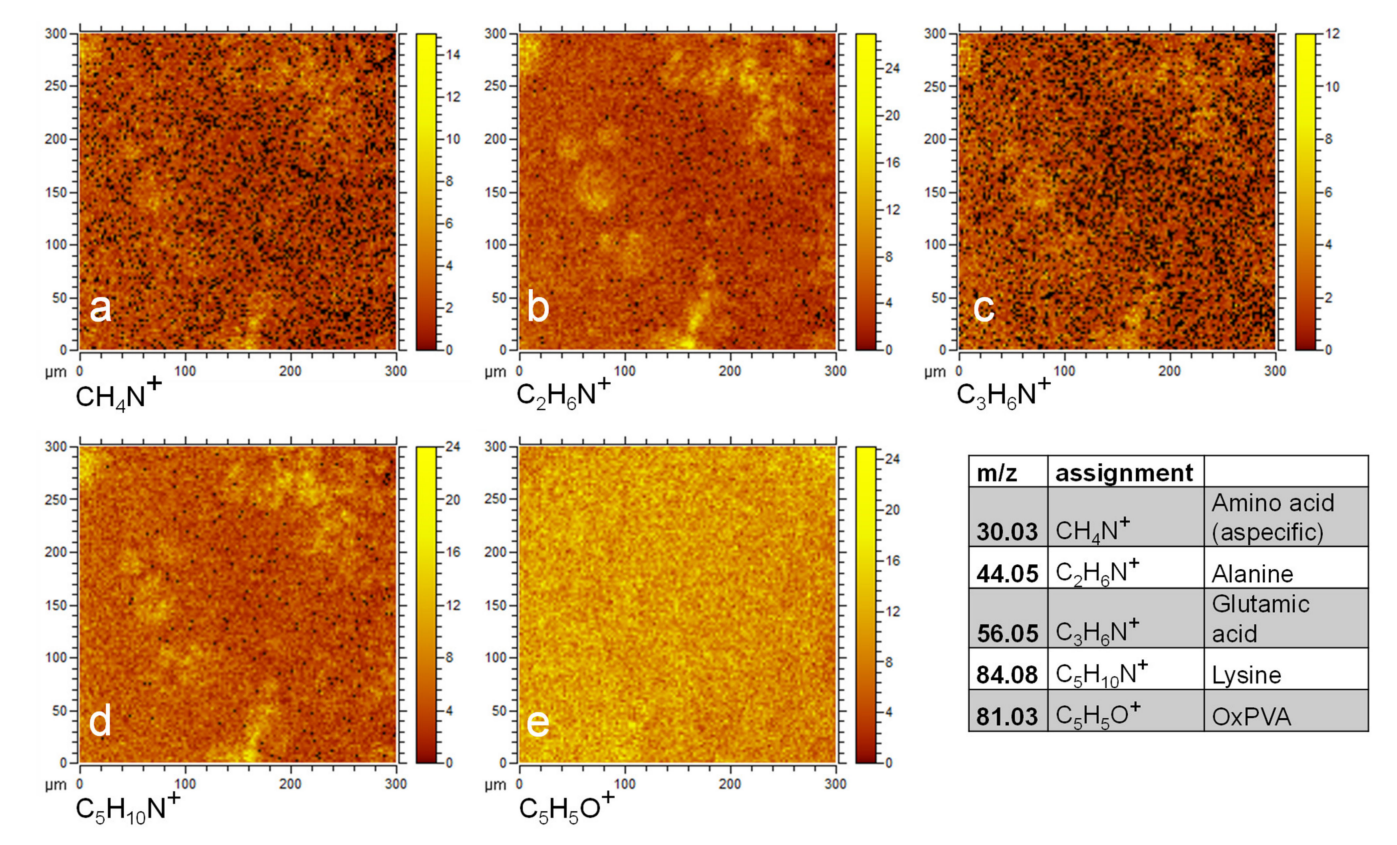
Chemical maps reporting the relative intensity of the peaks for the characteristic masses in the right side table (lateral resolution 5 µm): aspecific amino acid (**a**); alanine (**b**); glutamic acid (**c**); lysine (**d**); OxPVA (**e**).

Figure 5 shows the “chemical” maps of the surfaces, made by “mapping” the intensity of the peaks for the characteristic masses (lighter color, more intense peak). The maps show that the peptide distribution is quite uniform; the granules at the surface are indicative of EAK presence.

### 3.3. Absence of Cytoxicity Mediated by Functionalization and SH-SY5Y Cell Behavior on Scaffolds

Scaffolds in vitro cytocompatibility and cell behavior/organization after support seeding was evaluated by MTT assay and SEM analysis.

Preliminarily, an MTT assay showed no cytoxic effect of the decorated membranes on SH-SY5Y cells; after three days from seeding, the presence of cells was observed on all the samples. The functionalization approach itself (covalent binding and mechanical incorporation) also proved to be safe, as cell proliferation was observed on both OxPVA w/o IKVAV and w/o EAK.

**Figure 6:**
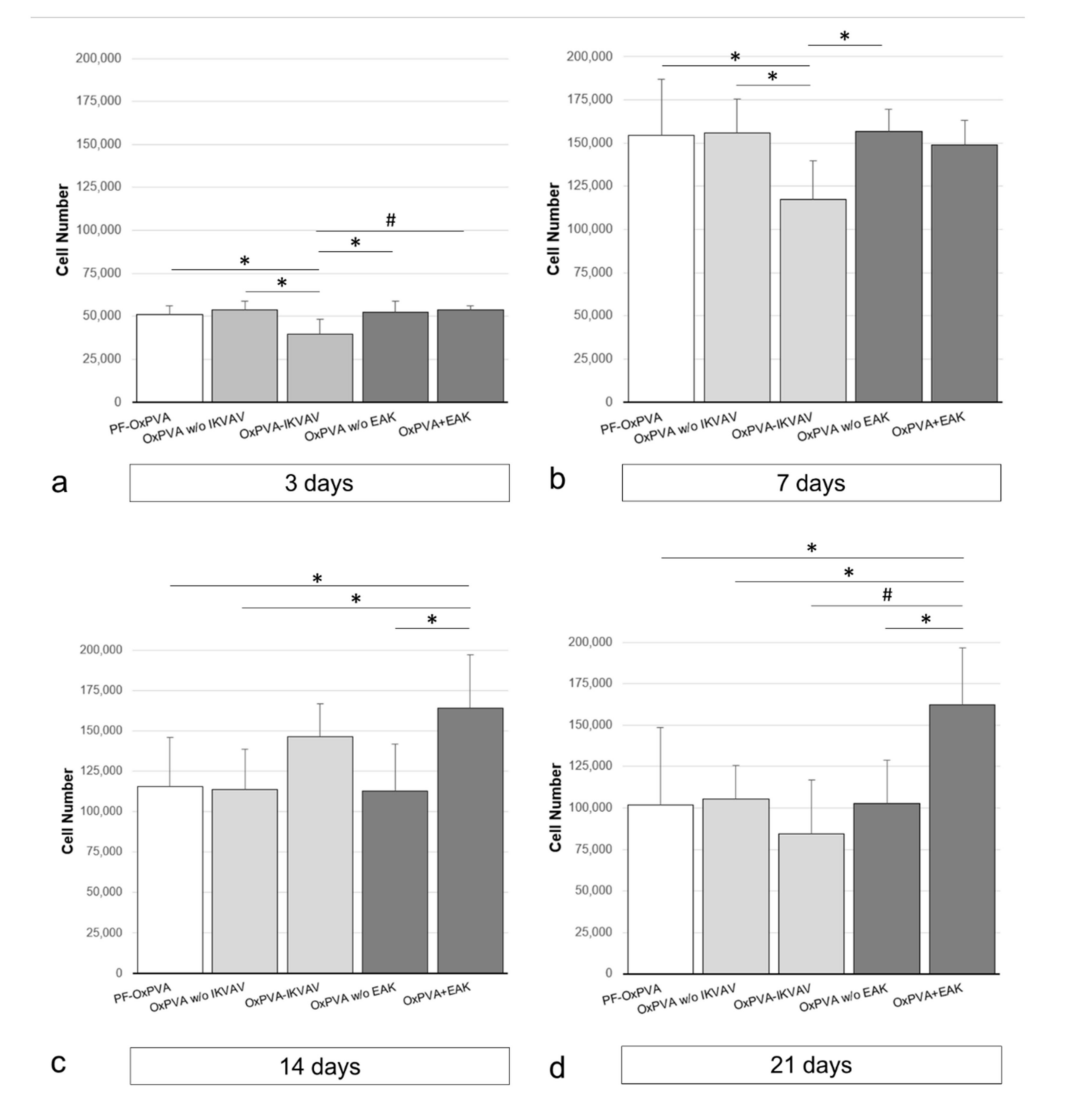
SH-SY5Y growth on patterned scaffolds. (**a**–**d**) MTT assay results show the different bioactive profile of the patterned scaffolds at 3, 7, 14 and 21 days from seeding (* *p*: <0.05; *# p*: <0.01). At the first time point, a significantly higher number of cells was detected on PF-OxPVA, OxPVA w/o IKVAV, OxPVA w/o EAK (*p*: <0.05) and OxPVA+EAK (<0.01) scaffolds versus OxPVA-IKVAV (**a**). After 7 days from seeding, a significantly higher number of proliferating cells was detected on PF-OxPVA, OxPVA w/o IKVAV and OxPVA w/o EAK scaffolds versus OxPVA-IKVAV (*p*: <0.05); conversely, no other statistically significant differences were observed (**b**). On day 14, OxPVA-EAK scaffolds assured a significantly higher number of cells (*p*: <0.05) compared to PF-OxPVA, OxPVA w/o IKVAV and OxPVA w/o EAK scaffolds; no other significant differences were observed (**c**). On day 21, the total cells number decreased on PF-OxPVA, OxPVA w/o IKVAV, OxPVA w/o EAK scaffolds and OxPVA-IKVAV scaffolds. Conversely, the OxPVA-EAK scaffolds showed a significantly higher cellular growth compared to the other groups (*p*: <0.05 versus PF-OxPVA, OxPVA w/o IKVAV, OxPVA w/o EAK and *p*: <0.01 versus OxPVA-IKVAV, respectively) (**d**).

Considering scaffold bioactivity, statistical differences were highlighted when comparing SH-SY5Y cell proliferation on supports. On day 3, non-bioactivated scaffolds (PF-OxPVA, OxPVA w/o IKVAV and w/o EAK) and OxPVA+EAK supports showed the presence of a significantly higher number of adherent cells versus OxPVA-IKVAV (*p*: <0.05 and <0.01, respectively). On the other hand, no statistical difference in sustaining cell adhesion/growth was detected among PF-OxPVA and OxPVA+EAK (Figure 6a). On day 7, a significantly higher bioactivity of control scaffolds (PF-OxPVA, OxPVA w/o IKVAV and w/o EAK) versus OxPVA-IKVAV (*p*: <0.05) was still highlighted by MTT assay; on the contrary, no difference was observed among OxPVA-IKVAV and OxPVA+EAK. Differences between PF-OxPVA and the other control groups (OxPVA w/o IKVAV and w/o EAK) versus OxPVA+EAK remained statistically insignificant (Figure 6b). As shown by the graphs, an increase in the total number of cells grown on scaffolds was demonstrated in all the experimental groups from day 3 to day 7. Subsequently, after 14 days from seeding, cell behavior was observed to be different according to the polymer support. In particular, on OxPVA-IKVAV and OxPVA+EAK scaffolds, an increase in total number of cells was detected, suggesting enhanced proliferation of SH-SY5Y populations on these supports; conversely, a reduction in total cellular elements was observed on the non-bioactivated supports (PF-scaffolds, OxPVA supports w/o IKVAV and w/o EAK) in comparison with day 7. At this end-point, the better results were assured by OxPVA-EAK, which promoted the growth of a significantly higher number of cells (*p*: <0.05) compared to PF-OxPVA, OxPVA supports w/o IKVAV and w/o EAK; no significant difference was statistically measured between OxPVA-IKVAV and OxPVA-EAK as well as versus the non-bioactivated supports (PF-scaffolds, OxPVA supports w/o IKVAV and w/o EAK) (Figure 6c). At the last end-point (21 days), total cell number still showed a slight decrease on PF-OxPVA scaffolds and supports w/o IKVAV and w/o EAK; the same behavior was displayed also by OxPVA-IKVAV, where reduction in proliferating cells was more appreciable. Interestingly, the OxPVA-EAK scaffolds sustained the growth of a significantly higher number of cells versus the other groups (*p*: <0.05 versus PF-OxPVA, OxPVA w/o IKVAV, OxPVA w/o EAK, and *p*: <0.01 versus OxPVA-IKVAV, respectively; Figure 6d).

**Figure 7:**
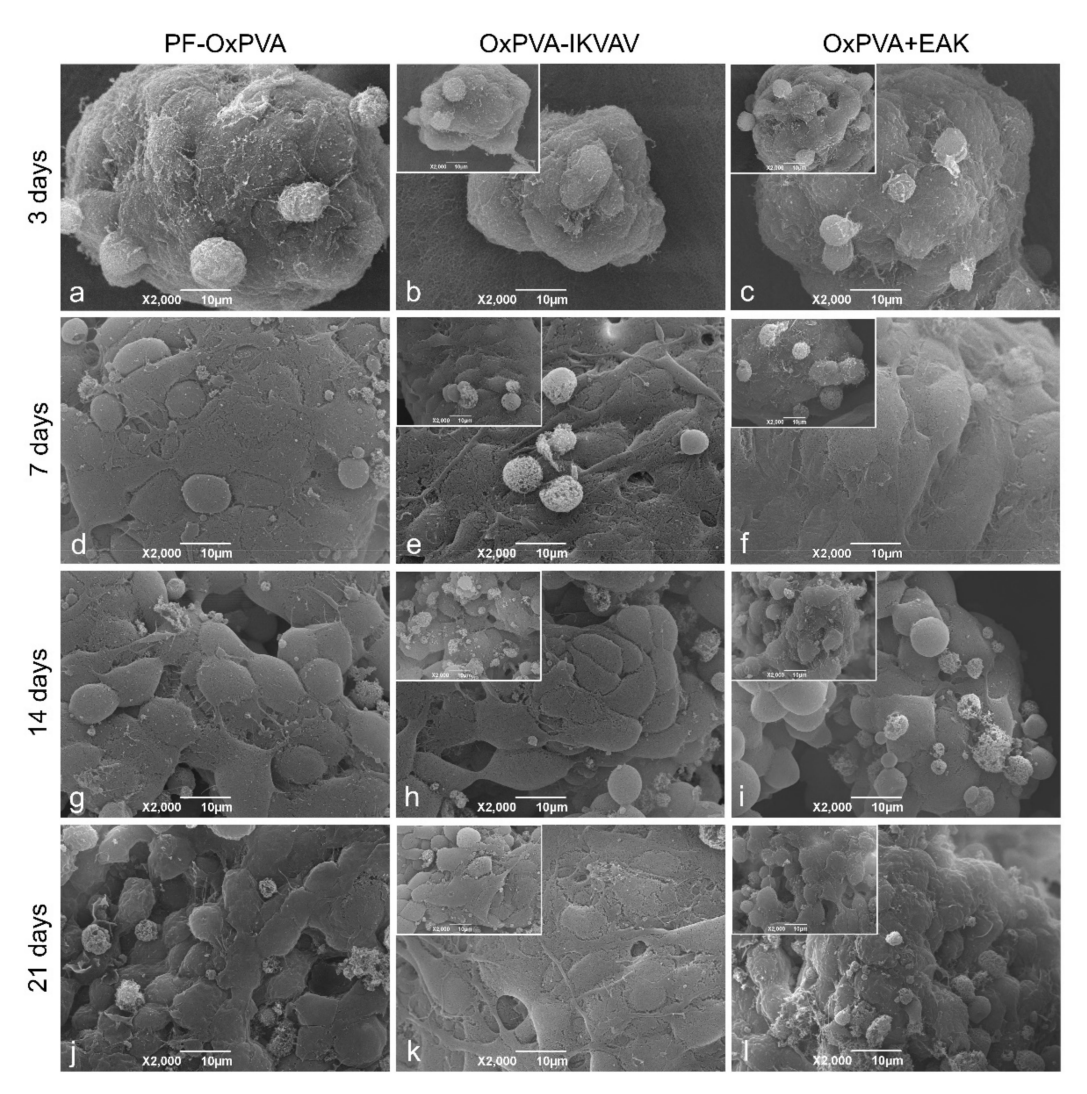
Evaluation by scanning electron microscopy of SH-SY5Y adhesion and proliferation on the patterned supports, at 3 (**a**–**c**), 7 (**d**–**f**), 14 (**g**–**i**) and 21 (**j**–**l**) days from seeding; inserts in (**b**,**e**,**h**,**k**) and (**c**,**f**,**i**,**l**) refer to cell adhesion and proliferation on the corresponding control groups, OxPVA w/o IKVAV supports and OxPVA w/o EAK scaffolds, respectively. Cells tend to form a progressively expanding monolayer upon scaffolds and they are clearly recognizable as proliferating elements on all the different supports here considered, at each time point. Scale bars: 10 µm.

SEM analysis highlighted the behavior of SH-SY5Y cells seeded on scaffolds. Adherent cells were observed in each group at each endpoint, supporting the MTT assay data. SH-SY5Y cell distribution on OxPVA w/o IKVAV and w/o EAK control scaffolds was similar to that displayed by PF-OxPVA samples (Figure 7).

## 4. Discussion

Since OxPVA development by partial oxidation, there was a consciousness of the great potential associated with this new synthetic polymer showing tunable mechanical behavior, swelling properties and biodegradability [5,14]. Additionally, another interesting feature related to the introduction of carbonyls in the OxPVA backbone is the possibility for its functionalization. The C=O groups can be involved in Schiff base reaction with the amino groups of growth factors, thus promoting OxPVA loading [5,7,15]. However, covalent binding of bioactive cues (proteins, growth factors, peptides) can also be pursued, in turn obtaining stable enriched platforms; attaching distinct biochemical motifs to polymeric supports is an intriguing method to select and recruit specific cell subsets within long nerve prothesis [16].

Peptides are recognized as important modifiers of polymer-based scaffolds. Their structural and biological properties sometimes overlap those of proteins, but they are simpler to synthetize, more cost-effective and resistant to environmental conditions (temperature, pH), as well as them presenting a lower immunogenic profile [17]. In addition, the synthetic approach offers the possibility of specific functional group insertion in peptide sequence, focused on peptide chemoselective conjugation to biomaterials.

IKVAV, the first peptide here considered, is a laminin-derived sequence, recognized by neural cells’ receptors. Together with the promotion of neural cell proliferation, it reduces the growth of fibroblasts [18,19]. In the perspective of OxPVA-IKVAV NCs fabrication and implantation, IKVAV presence could discourage the formation of excessive fibroconnective infiltrate after injury repair, also preventing neuromas [19]. In parallel, for the first time to our knowledge, the mechanical incorporation of a SAP (EAK) within a hydrogel was also assayed. SAPs are protein fragments self-organizing in amphiphilic β-sheet conformation by hydrogen bonds; SAPs are themselves hydrogels, highly stable in solution and with great potential in tissue engineering. Their specific nanofibrous structure, biomechanical properties and their ability to accelerate serum protein adsorption are promising features which could prompt peripheral nerve regeneration [9]. Another characteristic is their resistance to high temperature (90 °C) [9], an important feature considering that OxPVA requires being heated at 70 °C for pouring and assuring proper manipulation before FT. In this study, the SAP EAK was used as previously, demonstrating an attractive behavior towards enteric nervous system (ENS) cells [9].

The aim of covalent binding and mechanical incorporation of bio-cues is to create stable supporting platforms assuring a progressive superficial exposure of the bioactive sequences, along with polymer biodegradation. This approach is different than absorption which, taking advantage of OxPVA’s high swelling index, can sustain stimuli release for a protracted but limited time, and mainly encourages the initial phases of the regeneration process. Arguably, one strategy is not better than the others as what must be considered is the goal to be pursued.

Together with biochemical activation of the support, a physical stimulus (ultrastructural organization of the groove) was here included to optimize the performances of the scaffolds. For the first time, OxPVA showed ability in supporting cell adhesion/proliferation in vitro thanks to surface modification, even without biological cues. In previous studies, seeding attempts on smooth OxPVA supports were performed, however, combination with decellularized extracellular matrices was always required to trigger cell adhesion/colonization [5,20,21]. As reported for native PVA, it is likely that cell adherence is inhibited by the highly hydrophilic nature of the OxPVA hydrogel [10,22]. With the aim to solve this potential limit, inherent in the polymer intrinsic nature, the introduction of ultrastructural surface modification in OxPVA scaffolds manufacturing was here explored for the first time.

Surface microtopography is a tool to confer biomaterials’ bioactive properties and grow instructive signals (growth rate and orientation, migration, matrix proteins production), thus affecting peripheral nerve regeneration [23,24,25]. Many groove topographies exist and the identification of the most effective one can present a challenge; however, there is a consensus that conduits with longitudinally aligned inner textures can favorably modulate neuronal cell behavior. Additionally, a patterned groove also increases the surface-area-to-volume-ratio, promoting cells–scaffolds interactions, and ameliorating the microenvironment within the lumen because of an increased area for the dynamic diffusion of nutrients/waste products [26]. Another topic of discussion regards groove width. A 10–20 µm distance has been applied by many authors; however, it should be noticed that hierarchical nerve fascicles have a higher size (100 to 1000 μm) [23,26]. In the case of width of 10–20 μm, axons can grow in an improper manner with a reduction in NCs guidance function. NCs with higher inner microchannel width (200–300 μm) were distinguished for an effective guiding role towards axons, vascular cells and glial cells [27]; the same evidence was also displayed by Wang and collaborators [26] adopting the same topographies in hollow tubes with patterned inner walls. Here, a high width alligned pattern (300–400 μm) was used with encouaging results.

As reported in the literature, SH-SY5Y neuroblastoma cells share certain properties with primary neurons, thus being suitable for in vitro studies to mimic their attachment and proliferation [28,29,30]. The first step of the cell adhesion process is the cell-polymer interaction, which is crucial for proper tissue development/maintenance. However, different conditions may occur, including non-adhesion, passive adhesion and active adhesion [31]. If “non-adhesion” and “active adhesion” refer to attitudes with an immediate meaning, passive adhesion implies that cells attach easily but can also easily detach from surfaces. Considering the evidence gathered by the MTT assay, this is likely the behavior displayed by PF-OxPVA (as well as the other control groups) and OxPVA-IKVAV. These scaffold types sustained cell attachment, however, a diminished number of adherent cells was evidenced from day 7 onward for PF-OxPVA and the other control groups, with a higher reduction in the interval between day 7 and 14, followed by a slighter reduction from day 14 to 21. On the contrary, active adhesion was sustained by OxPVA+EAK revealing it as the most promising support among those here compared.

Control over groove microstructure was confirmed as fundamental to induce cell adhesion on the oxidized polymer; however, to boost the biological effect associated with it, the integration with biochemical cues is fundamental. This aspect was also confirmed by SH-SY5Y cells behavior on the control groups based on OxPVA w/o IKVAV and OxPVA w/o EAK, which showed a proliferation similar to that observed on PF-OxPVA supports. The comparable results displayed by the three control groups are explained by the fact that the functionalization methods (i.e., covalent binding and mechanical incorporation of the peptides) do not involve the use of molecules/reagents which may alter the chemical–physical properties of the polymer.

Comparing the two functionalization strategies, mechanical incorporation of the peptide probably allows for a more effective entrapment of the motif within the hydrogel mesh, not strictly depending on C=O sites (oxidation degree: 1% [5]) for IKVAV. Decoration of 2-dimensional or 3-dimensional supports by IKVAV is broadly recognized as a promising method to enhance cell adhesion, induce neural differentiation and promote nerve regeneration [32,33,34]; however, peptide bioactivity is affected by surface peptide density [35] that here might be too low, as it strictly depends on polymer carbonyl groups content (in our case, 1%). This hypothesis could therefore justify the unpromising results displayed by the OxPVA-IKVAV scaffolds. In parallel, mechanical incorporation of the SAP EAK, proving the better approach for OxPVA bioactivation, lays the basis for further investigation.

The limit of the study here presented is that it has not been provided comparative data on scaffolds ability in inducing cell differentiation. However, as mechanical incorporation distinguished over covalent binding, future investigations will focus on this strategy in detail. Different SAPs will be compared not only for their attitude towards cells adhesion and proliferation, but also including analyses for phenotype evaluation. For confirmation of in vitro data, preclinical studies in animal models of diseases will be required.

Smooth PF-OxPVA NCs, implanted to cover gaps of 5 mm in animal models of diseases, were distinguished in a previous study as guaranteeing a satisfactory outcome. The regenerated nerves within the conduit displayed a homogeneous distribution of myelinated/unmyelinated nerve fibers with an axon density (central portion) significantly higher than that of the reversed autograft [6]. It is expected that, similarly to Hsu and colleagues [36], when comparing microgrooved polylactic acid (PLA) conduits versus smooth PLA conduits, the patterning of OxPVA may further prompt peripheral nerve regeneration. In addition, the functionalization with bioactive cues (also in combination) may be adopted to confer specific characteristics to the prostheses, which may acquire the features of a smart next-generation device able to optimize axons regeneration and discourage fibro-connective infiltration, thus optimizing nerve regeneration and function recovery.

Better outcomes in injured nerves’ function/sensitivity recovery are assured by therapies allowing for short regeneration times. Thus, in the perspective of future clinical use of on-the-bench bioactivated OxPVA NCs, supportive therapies, as in photobiomodulation, may also be adopted for fully satisfactory outcomes. Combining effective next-generation nerve guides with low-level laser therapies, assuring for improvement of the nerve regeneration process, assistance in muscular reinnervation, decrease in inflammatory cytokine levels and pain [37] may further boost the regenerative process, counteracting retrograde axonal degeneration with a prompt re-establishment of peripheral nerve continuity.

## 5. Conclusions

According to the study results, groove-patterned OxPVA scaffolds, decorated with EAK, display promising characteristics for enriched devices development. Mechanical incorporation of SAPs seems to be intriguing, distinguished as an easy and fast approach for effective NCs functionalization.

Future studies will be undertaken to compare other biochemical motifs’ potential to promote injured peripheral nerve recovery. In particular, the arrangement of complex patterned devices, showing the presence of different sequences mixed in percentage, may represent a bright perspective to finely control the peripheral nerve regenerative process.

## Data Availability

Not applicable.

## Supplementary Materials

The following are available online at www.mdpi.com/article/10.3390/polym13193372/s1, Figure S1: Analytical chromatogram of purified Aoa-xx-IKVAV obtained under the following conditions: column, Jupiter C_18_ (5 μm, 300 Å, 4.6 × 250 mm^2^); injected volume, 200 μL; flow rate, 0.5 mL/min; detector wavelength, 214 nm; eluent A, 0.05% TFA in MilliQ water; eluent B, 0.05% TFA in 2-proplanol/water (40/60); gradient, from 40% to 55% of eluent B in 30 min, Figure S2: ESI-ToF mass spectrum of purified Aoa-xx-IKVAV. The mass analysis confirmed the presence of the target peptide (theoretical weight: 855.06 Da, experimental weight: 855.49 Da), Figure S3: Analytical chromatogram of purified EAK obtained under the following conditions: column, NovaPak HR C18 (4 μm, 60 Å, 3.9 × 300 mm^2^); injected volume, 150 μL; flow rate, 1 mL/min; detector wavelength, 214 nm; eluent A, 0.05% TFA in MilliQ water; eluent B, 0.05% TFA in CH_3 CN; gradient, from 13% to 33% of eluent B in 20 min, Figure S4: MALDI mass spectrum of purified EAK. The mass analysis confirmed the presence of the target peptide (theoretical weight: 1614.79 Da, experimental weight: 1614.50 Da).
