# Supplementary material for: Bioactivated Oxidized Polyvinyl Alcohol towards Next-Generation Nerve Conduits Development
Source: Polymers (Basel). 2021 Sep 30;13(19):3372. doi: 10.3390/polym13193372 (PMC8512895; doi:10.3390/polym13193372)
Supplement: Supplementary file 1 [file polymers-13-03372-s001.zip › polymers-1348681-SM.pdf]

Supplementary Material

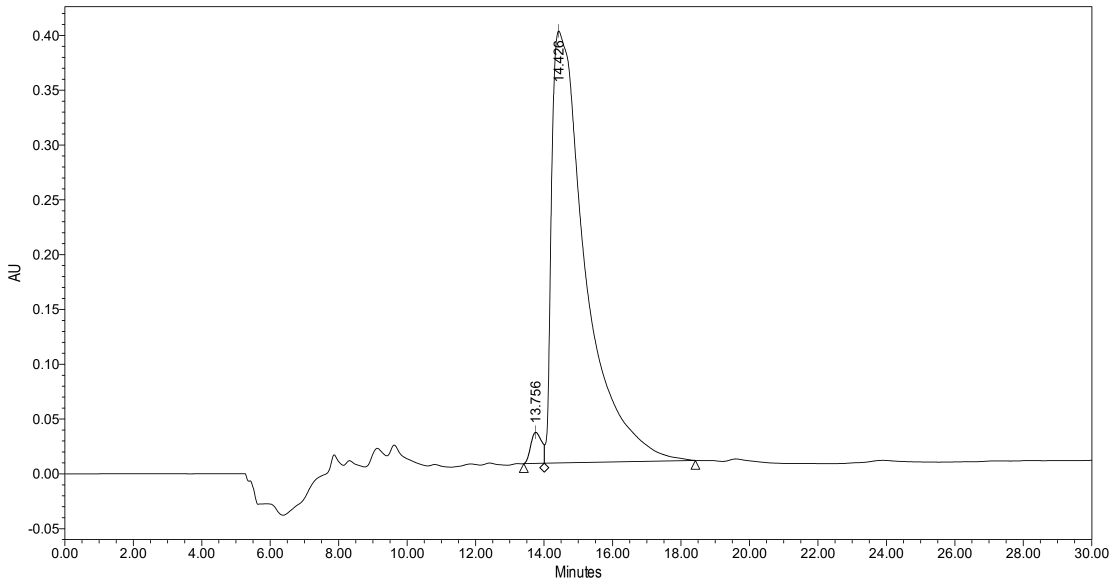

Figure S1. Analytical chromatogram of purified Aoa-xx-IKVAV obtained under the following conditions: column, Jupiter C_{18} (5 µm, 300 Å, 4.6 mm × 250 mm); injected volume, 200 µL; flow rate, 0.5 mL/min; detector wavelength, 214 nm; eluent A, 0.05% TFA in MilliQ water; eluent B, 0.05% TFA in 2-proplanol/water (40/60); gradient, from 40% to 55% of eluent B in 30 minutes.

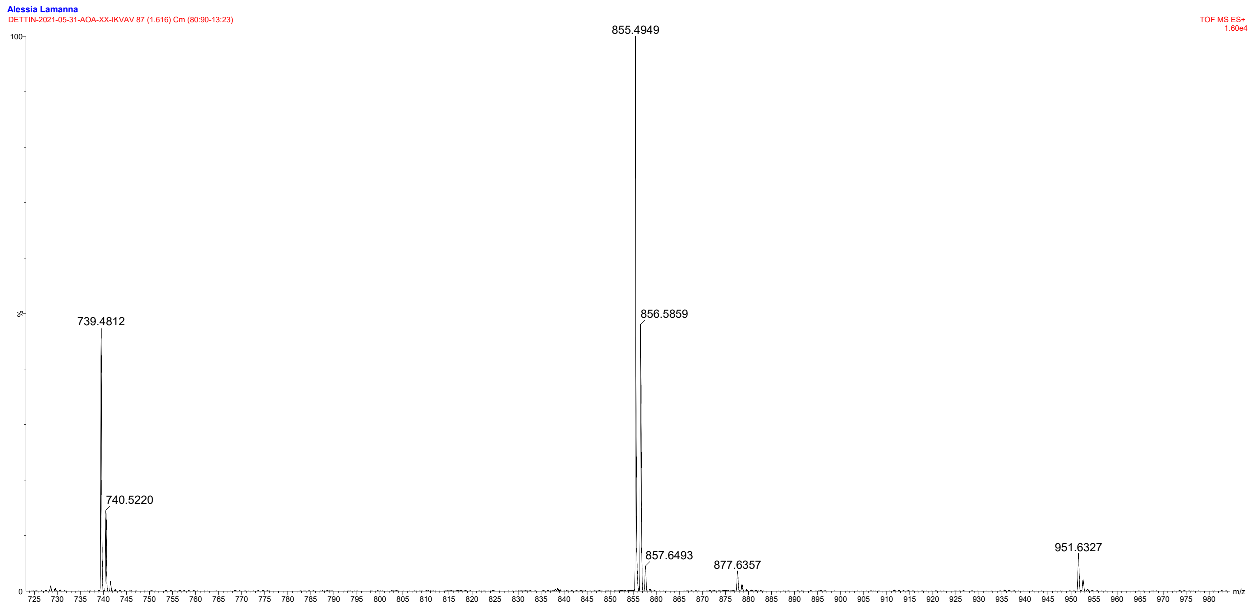

Figure S2. ESI-ToF mass spectrum of purified Aoa-xx-IKVAV. The mass analysis confirmed the presence of the target peptide (theoretical weight: 855.06 Da, experimental weight: 855.49 Da).

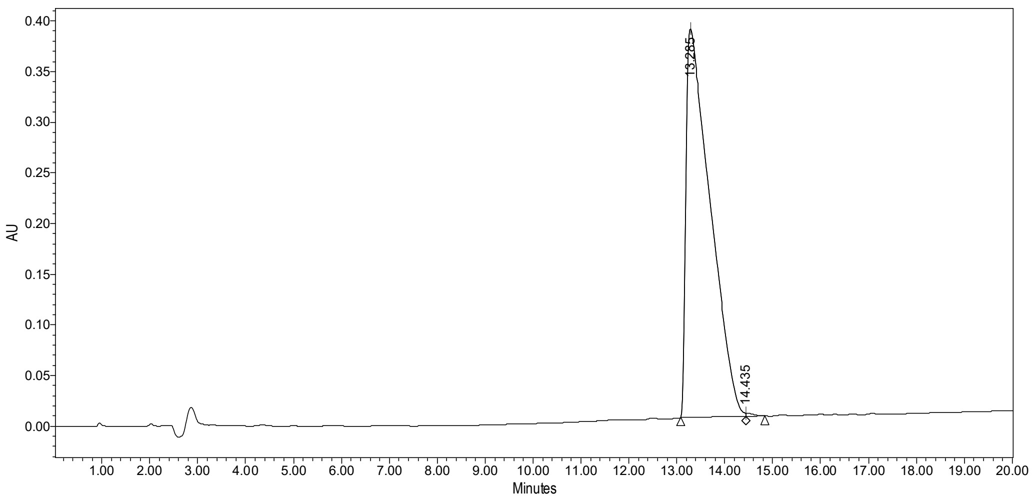

Figure S3. Analytical chromatogram of purified EAK obtained under the following conditions: column, NovaPak HR C18 (4 μm, 60 Å, 3.9 mm × 300 mm); injected volume, 150 μL; flow rate, 1 mL/min; detector wavelength, 214 nm; eluent A, 0.05% TFA in MilliQ water; eluent B, 0.05% TFA in CH_3CN; gradient, from 13% to 33% of eluent B in 20 minutes.

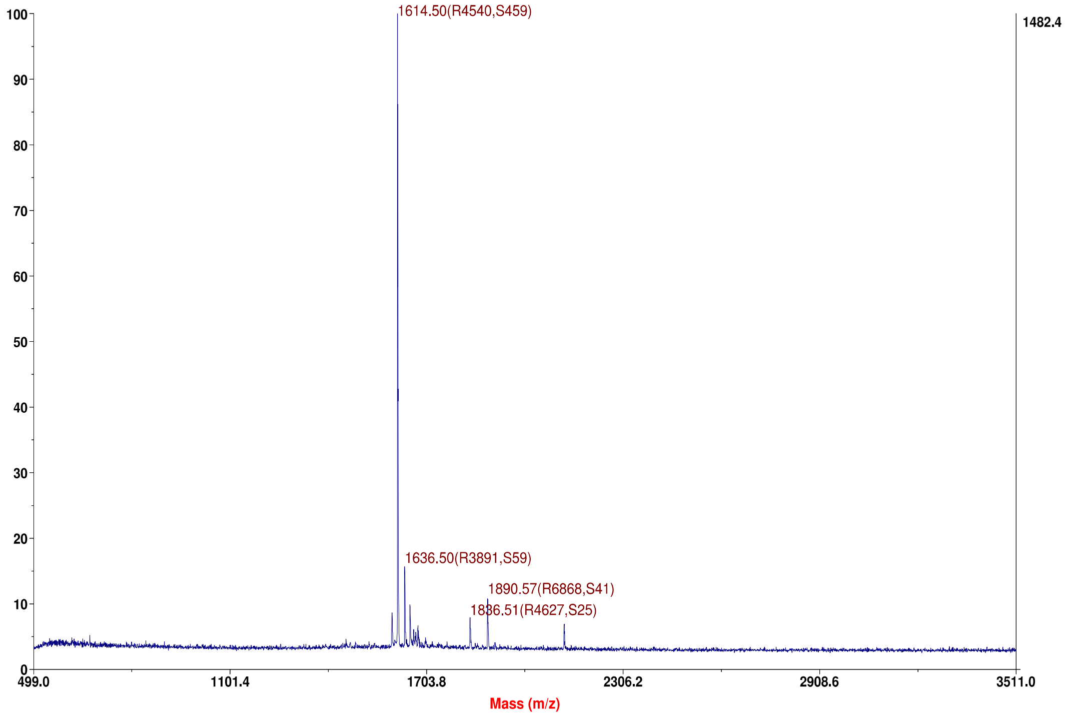

Figure S4. MALDI mass spectrum of purified EAK. The mass analysis confirmed the presence of the target peptide (theoretical weight: 1614.79 Da, experimental weight: 1614.50 Da).

Table S1. XPS results.

Sample	Signal	Assignment	BE (eV)	FWHM (eV)	Atomic ratios (%)
**PF-OxPVA**	C1s	C–C	285.0	1.53	54
		C–N	286.1		27
		C–O	287.5		11
		C=O	288.9		6
		COOH	290.3		2
	O1s	C=O	531.0	2.05	53
		C–O	532.5		23
			533.5		19
		H_2O	535.0		5
**OxPVA-IKVAV**	C1s	C–C	285.0	1.42	52
		C–N	286.0		28
		C–O	287.2		12
		C=O	288.7		5
		COOH	289.7		3
	O1s	C=O	531.3	2.06	58
		C–O	533.0		35
		H_2O	534.7		7
	N1s	C–N	400.7	2.55	100
